# Supplementary material for: Dual functionalized brain-targeting nanoinhibitors restrain temozolomide-resistant glioma via attenuating EGFR and MET signaling pathways
Source: Nat Commun. 2020 Jan 30;11:594. doi: 10.1038/s41467-019-14036-x (PMC6992617; doi:10.1038/s41467-019-14036-x)
Supplement: Supplementary file 1 — Supplementary Information [file 41467_2019_14036_MOESM1_ESM.pdf]

SUPPLEMENTARY INFORMATION

Dual functionalized brain-targeting nanoinhibitors restrain temozolomide resistant glioma via attenuating EGFR and MET signaling pathways

Meng et al.

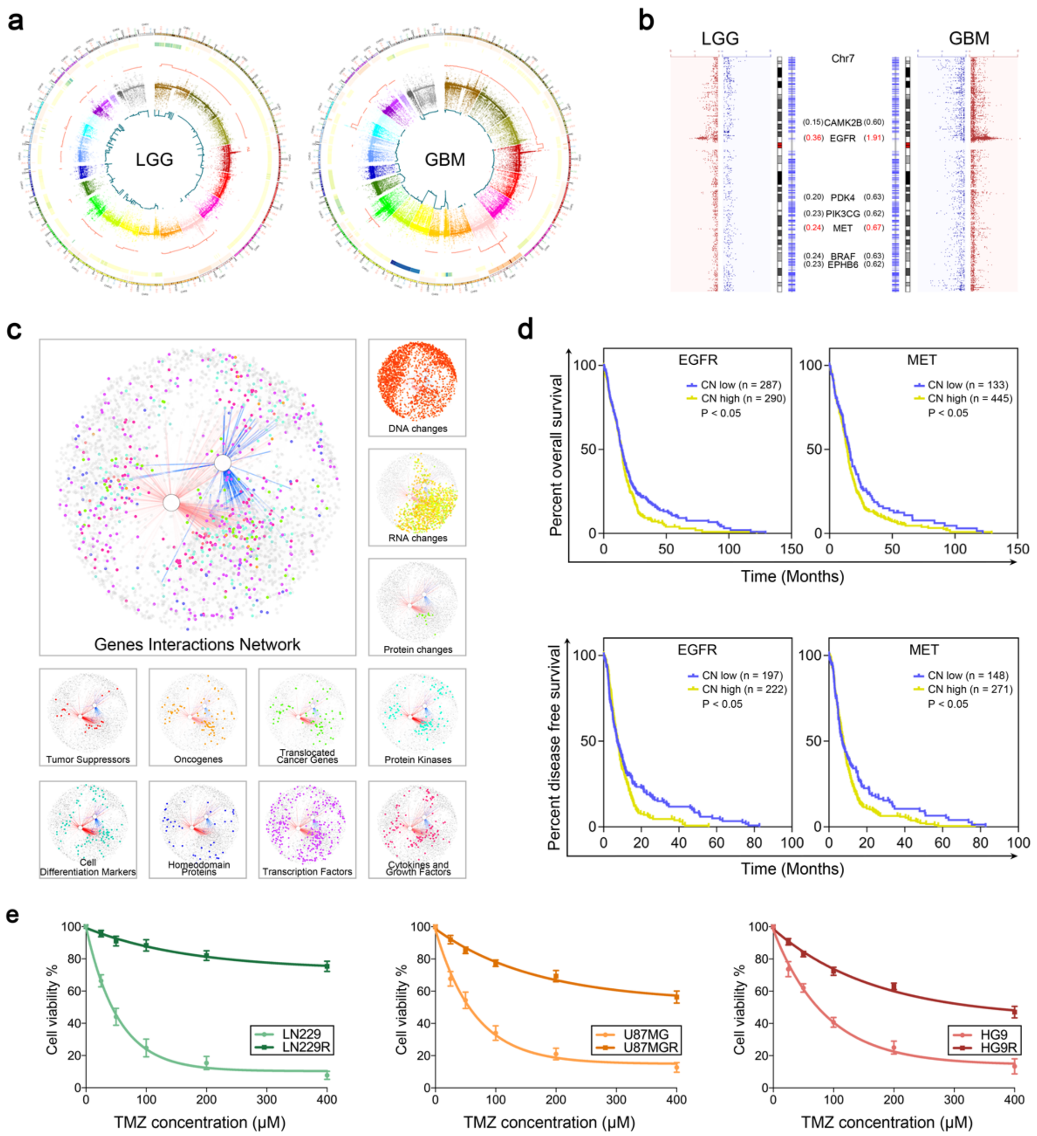

Supplementary Figure 1. The amplifications of EGFR and MET are associated with many genomic, transcriptomic and proteomic changes in gliomas.

(a) The genomic copy number variations of LGG and GBM. **(b)** The top seven amplification of RTK genes in LGG and GBM. **(c)** The EGFR and MET amplifications were simultaneously associated with a number of genomic variations, differential gene expression and protein profiling regulation. **(d)** The overall survival and progression free survival of patients with different EGFR and MET copy numbers. P value is determined by log-rank test. **(e)** The IC50 of TMZ in LN229, LN229R, U87MG, U87MGR, HG9 and HG9R (n = 6). The error bars represent the S.D. of six measurements.

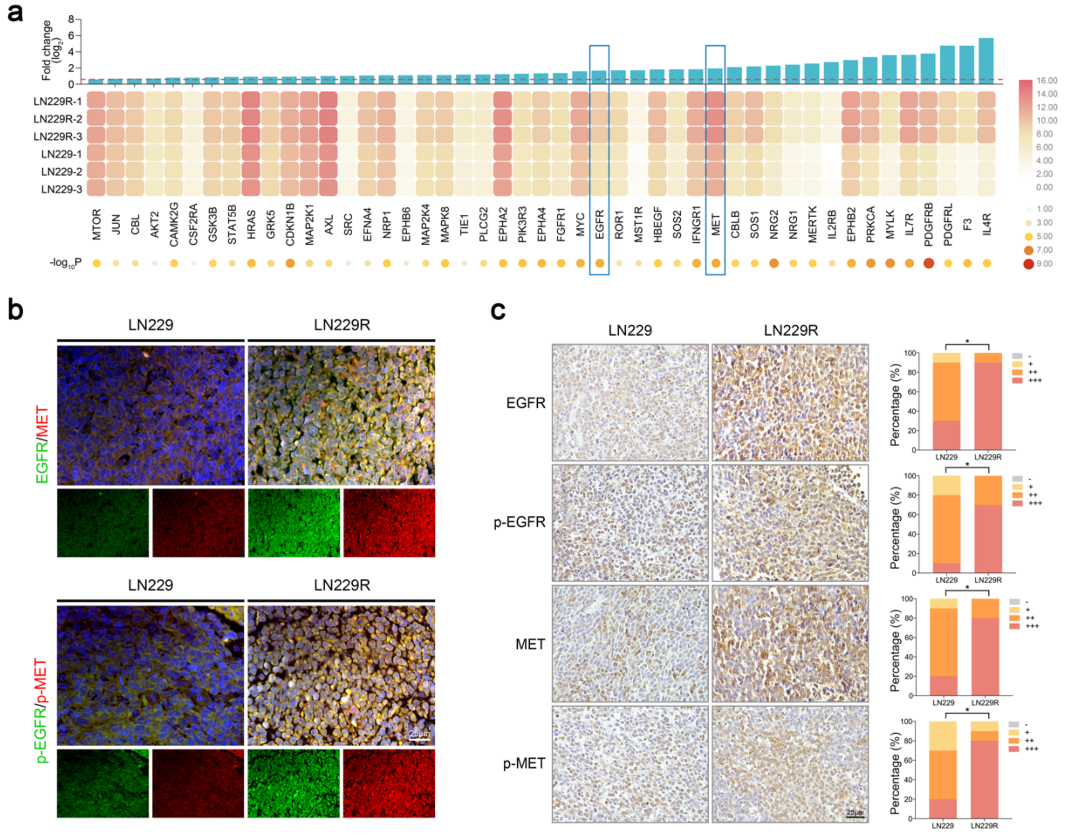

Supplementary Figure 2. The overactivation of EGFR and MET signaling pathways in TMZ resistant gliomas.

(a) The differential expressed RTK genes between LN229 and LN229R cells. **(b)** The immunofluorescence assay of EGFR, MET, p-EGFR and p-MET in glioma tissues derived from LN229 and LN229R, respectively. Scale bar = 25 μm. **(c)** The immunohistochemistry assay of EGFR, MET, p-EGFR and p-MET in glioma tissues derived from LN229 and LN229R, respectively. Scale bar = 25 μm. P value is determined by Chi-squared test. Significant results are presented as *P < 0.05.

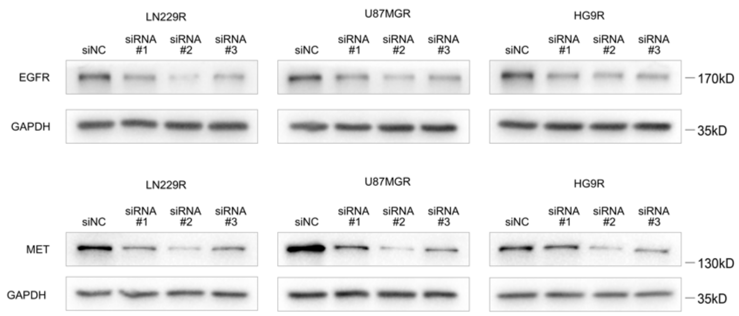

Supplementary Figure 3. The validation of siRNAs targeting EGFR and MET by Western blot.

The Western blot validation of EGFR and MET knocked down by siRNAs.

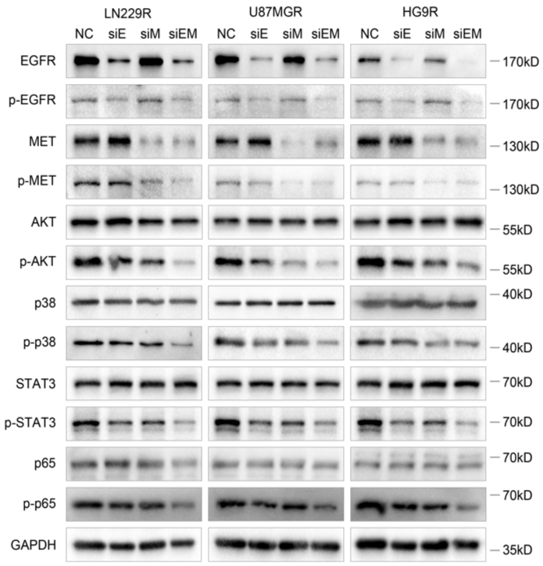

Supplementary Figure 4. The protein expression of EGFR and MET signaling pathways with siRNAs targeting EGFR or MET.

The Western blot assay of protein expression of EGFR and MET signaling pathways with siRNAs targeting EGFR or MET.

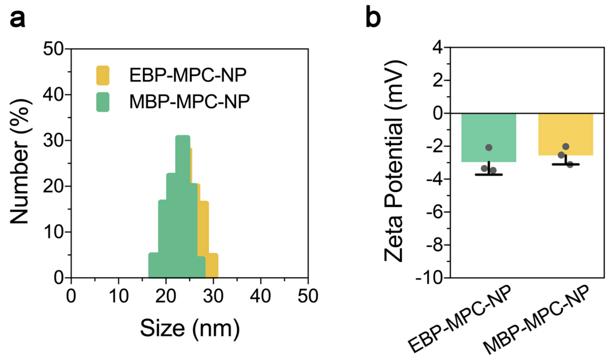

Supplementary Figure 5. The physical characteristics of EBP-MPC-NP and MBP-MPC-NP. (a) The dynamic light scattering (DLS) measurements of EBP-MPC-NP and MBP-MPC-NP. **(b)** The zeta potential data of EBP-MPC-NP and MBP-MPC-NP (n = 3). The error bars represent the S.D. of three measurements.

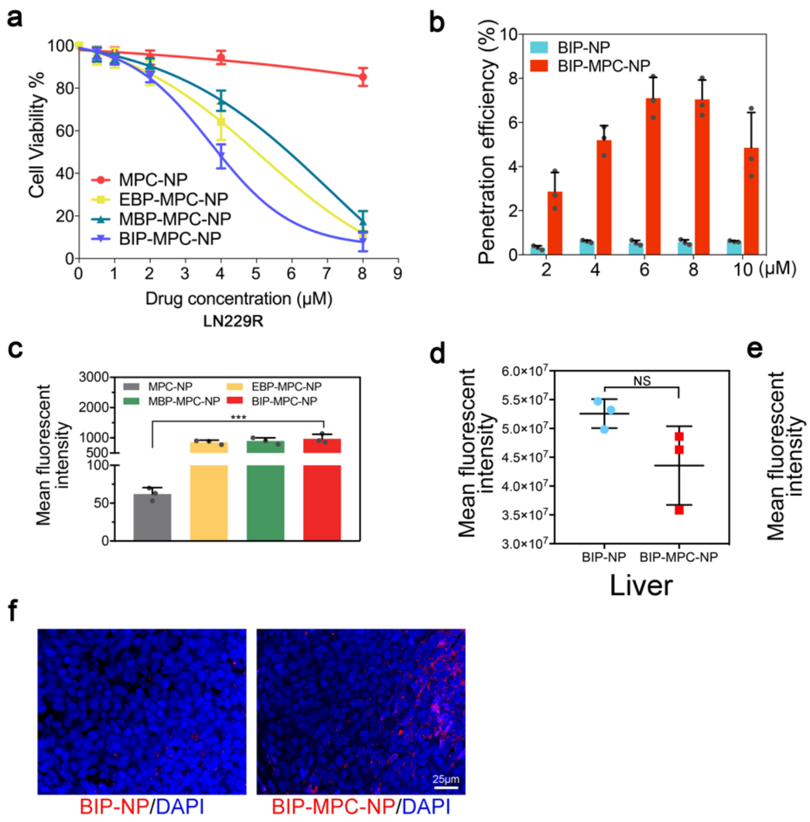

Supplementary Figure 6. The distribution of nanoinhibitors after penetrating BBB models *in vitro* and *in vivo*.

(a) The IC50 of MPC-NP, EBP-MPC-NP, MBP-MPC-NP and BIP-MPC-NP at 24 hours in LN229R (n = 6). **(b)** The transportation assays showed the permeability of BIP-NP and BIP-MPC-NP with different concentrations *in vitro*. **(c)** The flow cytometric analysis of cells with MPC-NP, EBP-MPC-NP, MBP-MPC-NP or BIP-MPC-NP after penetrating BBB model *in vitro* (n = 3). **(d-e)** The mean fluorescent intensity of BIP-NP and BIP-MPC-NP distributing in liver and kidney (n = 3). **(f)** The tumor tissue sliced distributions indicating a higher penetration of BIP-MPC-NP than that of BIP-NP within tumors. Scale bar = 25 μm. The error bars represent the S.D. of three or six measurements. P value is determined by Student's *t*-test. Significant results are presented as NS non-significant, ***$P < 0.001$.

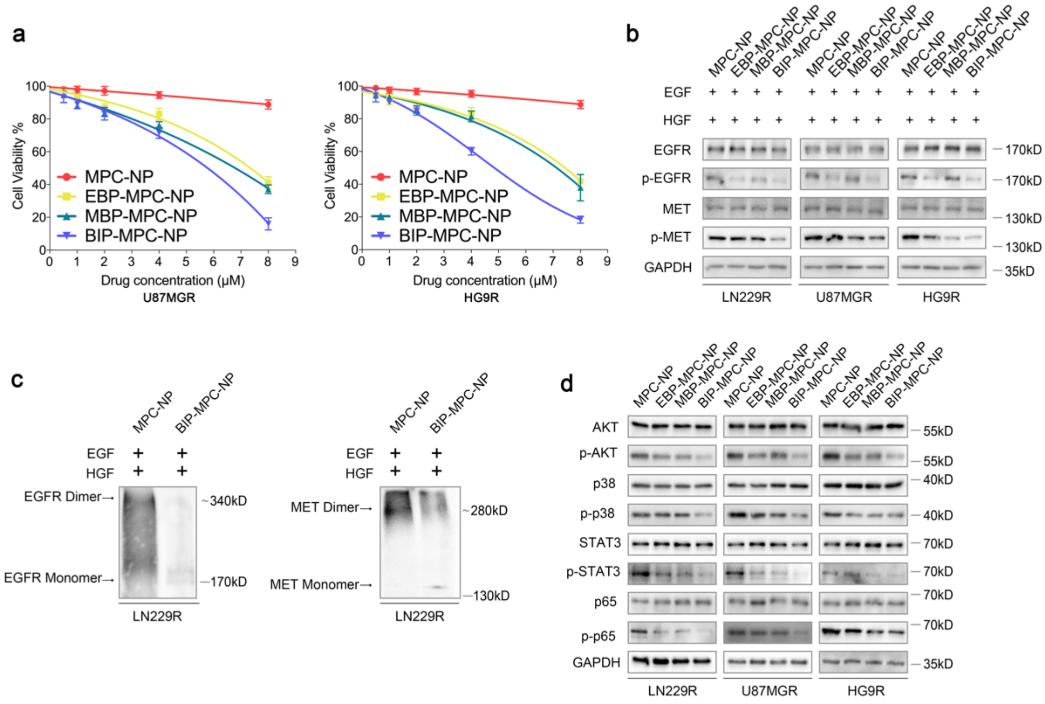

Supplementary Figure 7. The effect and the specificity of nanoinhibitors by Western blot.

(a) The IC50 of MPC-NP, EBP-MPC-NP, MBP-MPC-NP and BIP-MPC-NP at 24 hours in U87MGR and HG9R (n = 6). The error bars represent the S.D. of six measurements. **(b)** The EGFR, p-EGFR, MET and p-MET expression in TMZ resistant glioma cells treated with different nanoinhibitors in EGF and HGF dependent manners. **(c)** The levels of EGFR and MET dimers in TMZ resistant glioma cells treated with BIP-MPC-NP and with EGF and HGF. **(d)** The protein levels of EGFR and EMT signaling pathways in TMZ resistant glioma cells treated with nanoinhibitors.

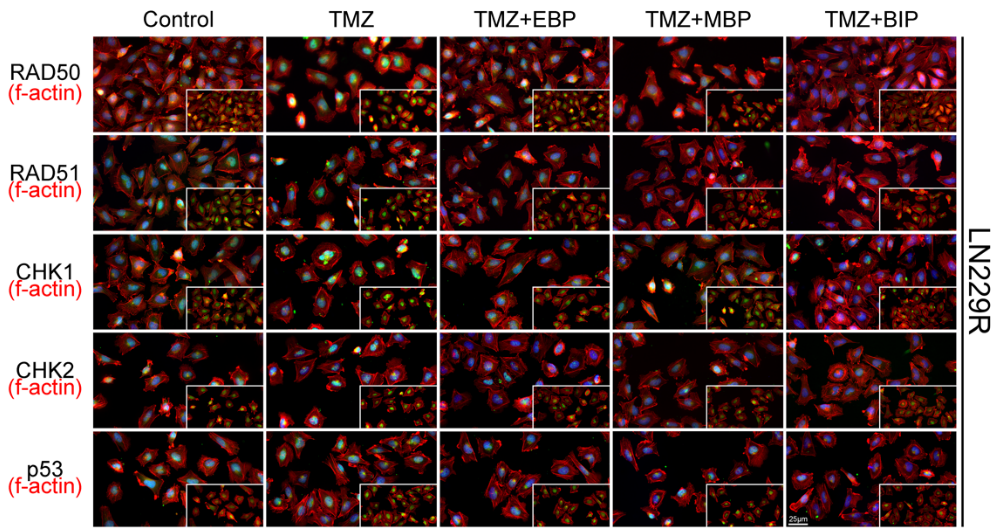

Supplementary Figure 8. The immunofluorescence of DNA damage repair modules in LN229R treated with TMZ and different nanoinhibitors.

The immunofluorescence of RAD50, RAD51, CHK1, CHK2 and p53 in LN229R treated with TMZ and different nanoinhibitors. Scale bar = 25 μm.

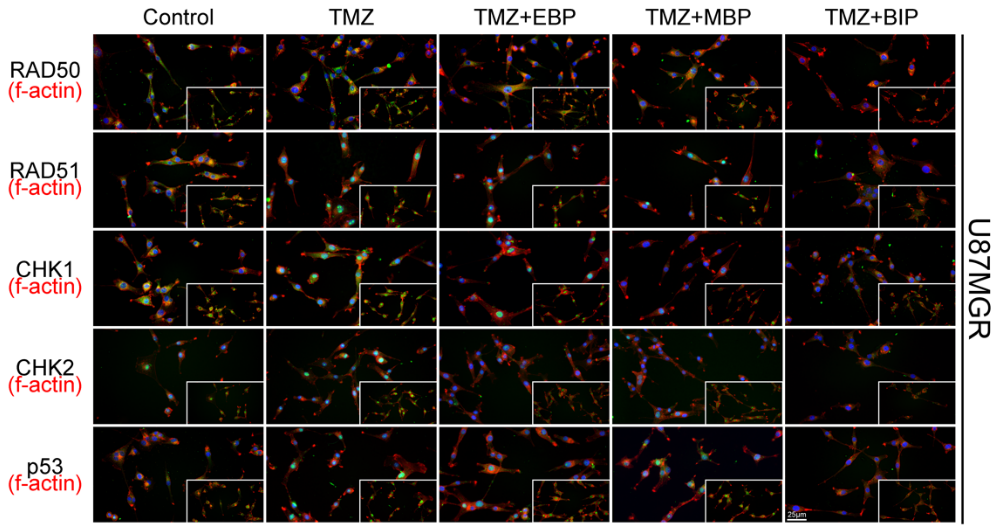

Supplementary Figure 9. The immunofluorescence of DNA damage repair modules in U87MGR cells treated with TMZ and different nanoinhibitors.

The immunofluorescence of RAD50, RAD51, CHK1, CHK2 and p53 in U87MGR treated with TMZ and different nanoinhibitors. Scale bar = 25 μm.

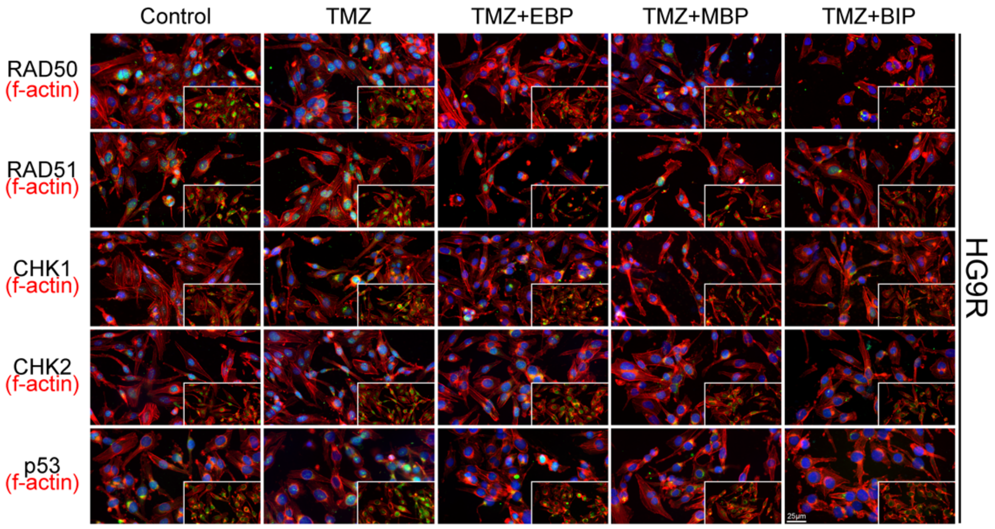

Supplementary Figure 10. The immunofluorescence of DNA damage repair modules in HG9R cells treated with TMZ and different nanoinhibitors.

The immunofluorescence of RAD50, RAD51, CHK1, CHK2 and p53 in HG9R treated with TMZ and different nanoinhibitors. Scale bar = 25 μm.

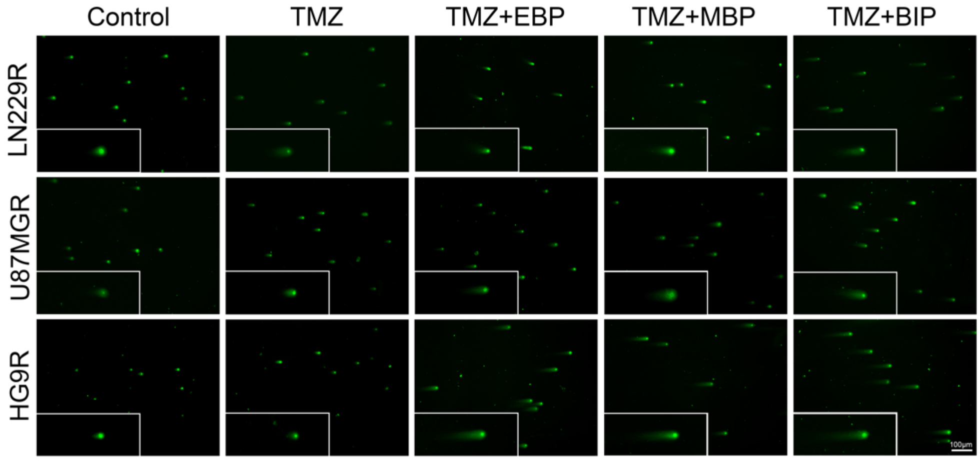

Supplementary Figure 11. The comet assay of TMZ resistant glioma cells with TMZ and different nanoinhibitors.

The comet assay of LN229R, U87MGR and HG9R treated with TMZ and different nanoinhibitors. Scale bar = 100 μm.

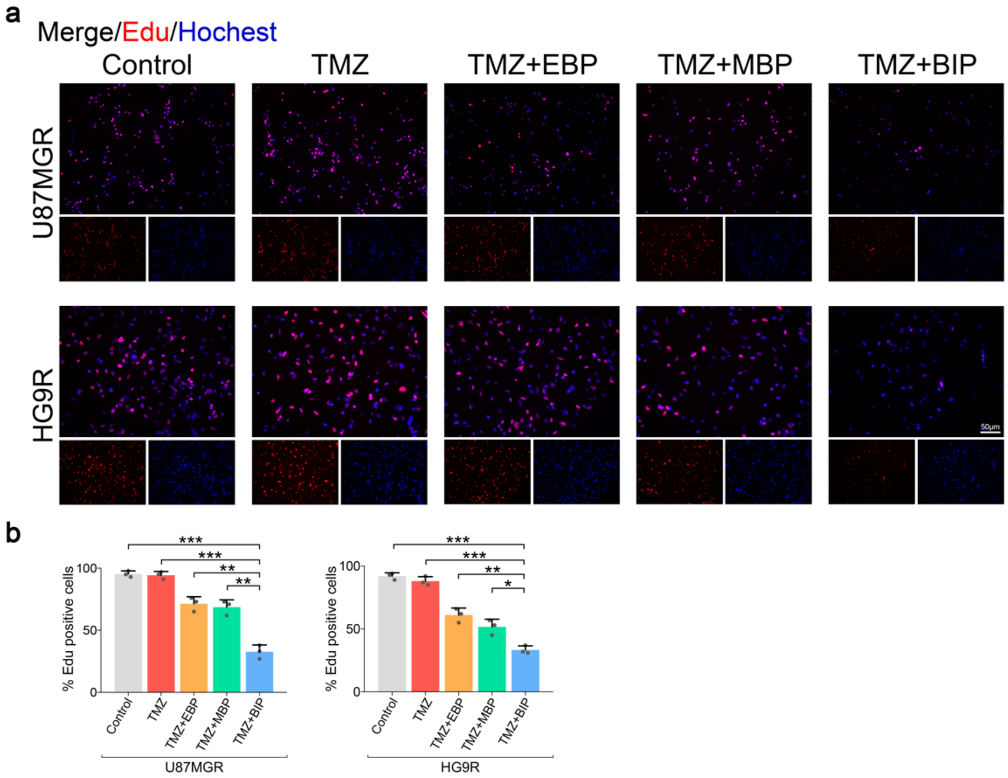

Supplementary 12. The EdU assays of U87MGR and HG9R treated with TMZ and different nanoinhibitors.

(a) The EdU assays of U87MGR and HG9R treated with TMZ and different nanoinhibitors. Scale bar = 50 µm. **(b)** The statistic of the EdU assays (n = 3). The error bars represent the S.D. of three measurements. P value is determined by Student's *t*-test. Significant results are presented as *P < 0.05, **P < 0.01, ***P < 0.001.

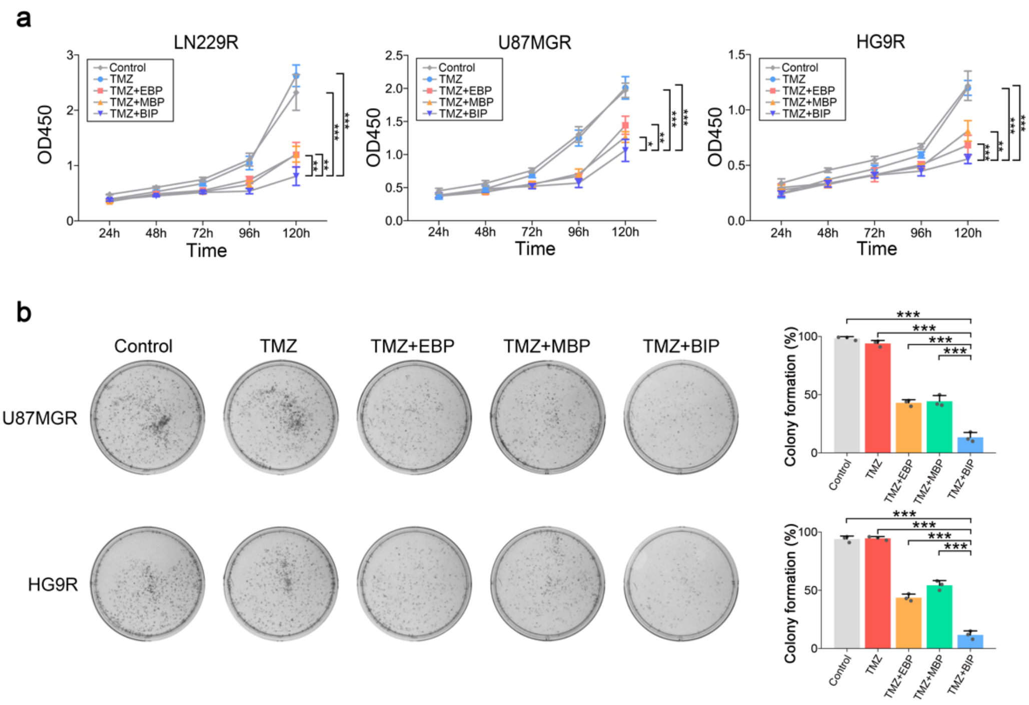

Supplementary Figure 13. The proliferation inhibition of cells treated with TMZ and different nanoinhibitors.

(a) The CCK-8 proliferation assays of LN229R, U87MGR and HG9R cells treated with TMZ and different nanoinhibitors (n = 6). **(b)** The colony formation assay of U87MGR and HG9R cells treated with TMZ and different nanoinhibitors. The histogram displayed the statistics of colony formation assays (n = 3). The error bars represent the S.D. of three or six measurements. P value is determined by Student's *t*-test. Significant results are presented as *P < 0.05, **P < 0.01, ***P < 0.001.

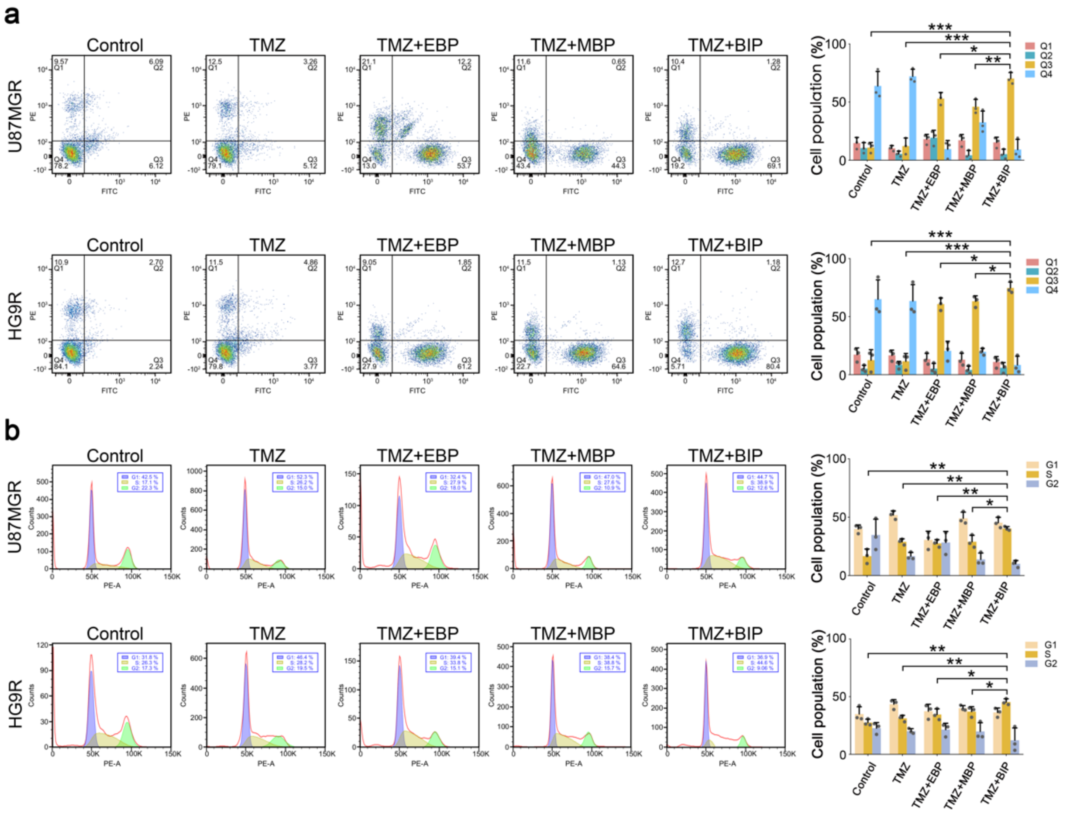

Supplementary Figure 14. The cell apoptosis assay and cell cycle analysis of U87MGR and HG9R cells treated with TMZ and different nanoinhibitors.

(a) The cell apoptosis assay of U87MGR and HG9R cells treated with TMZ and different nanoinhibitors and the statistic in histogram (n = 3). **(b)** The cell cycle analysis of U87MGR and HG9R cells treated with TMZ and different nanoinhibitors and the statistics in histogram (n = 3). The error bars represent the S.D. of three measurements. P value is determined by Student's *t*-test. Significant results are presented as *P < 0.05, **P < 0.01, ***P < 0.001.

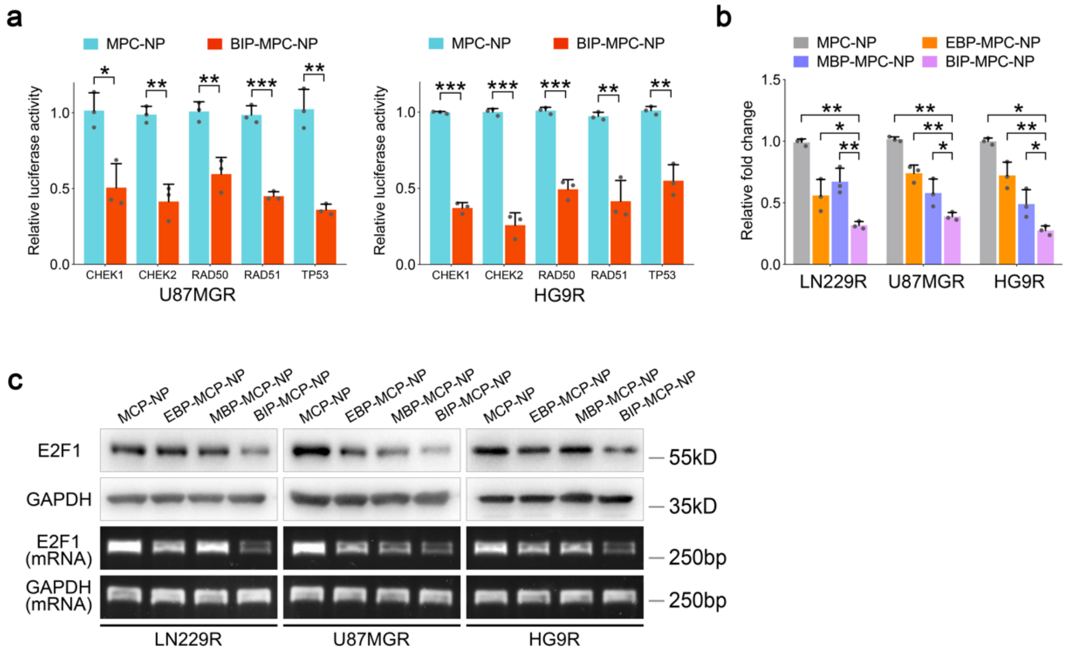

Supplementary Figure 15. The inhibition of *E2F1* expression and transcriptional activity on DNA damage repair modules by nanoinhibitors.

(a) The luciferase reporter assays representing the different E2F1 transcriptional activity on the promoter regions of *CHEK1*, *CHEK2*, *RAD50*, *RAD51* and *TP53* in U87MGR and HG9R cells treated with MPC-NP or BIP-MPC-NP (n = 3). **(b)** The E2F1 mRNA level in LN229R, U87MGR and HG9R treated with different nanoinhibitors (n = 3). **(c)** The E2F1 protein expression and mRNA expression in LN229R, U87MGR and HG9R treated with different nanoinhibitors. The error bars represent the S.D. of three measurements. P value is determined by Student's *t*-test. Significant results are presented as *P < 0.05, **P < 0.01, ***P < 0.001.

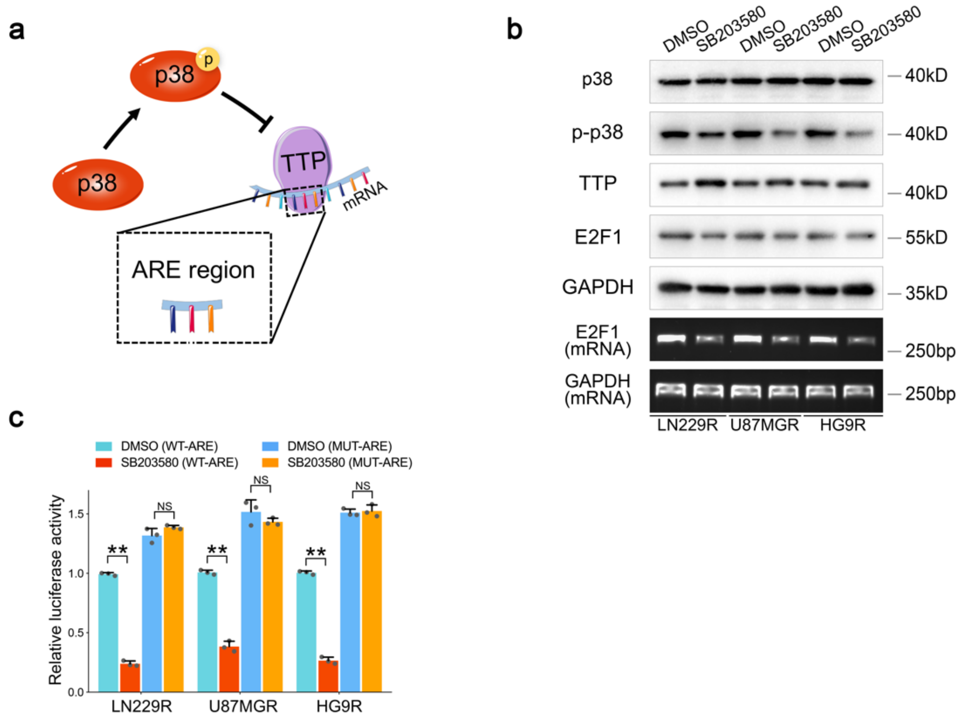

Supplementary Figure 16. The regulation of E2F1 containing ARE motifs by p-p38 mediated TTP.

(a) The illustration depicting the regulation of E2F1 containing ARE motifs by p-p38 mediated TTP. **(b)** The expression of p38, p-p38, TTP and E2F1 (Western blot) and the E2F1 mRNA (PCR) in TMZ resistant glioma cells treated with SB203580. **(c)** The inhibitory effect of TTP on wild type or mutant type ARE motifs within the E2F1 mRNA 3'-UTR in TMZ resistant glioma cells treated with SB230580 (n = 3). The error bars represent the S.D. of three measurements. P value is determined by Student's *t*-test. Significant results are presented as NS non-significant, $^{**}P < 0.01$.

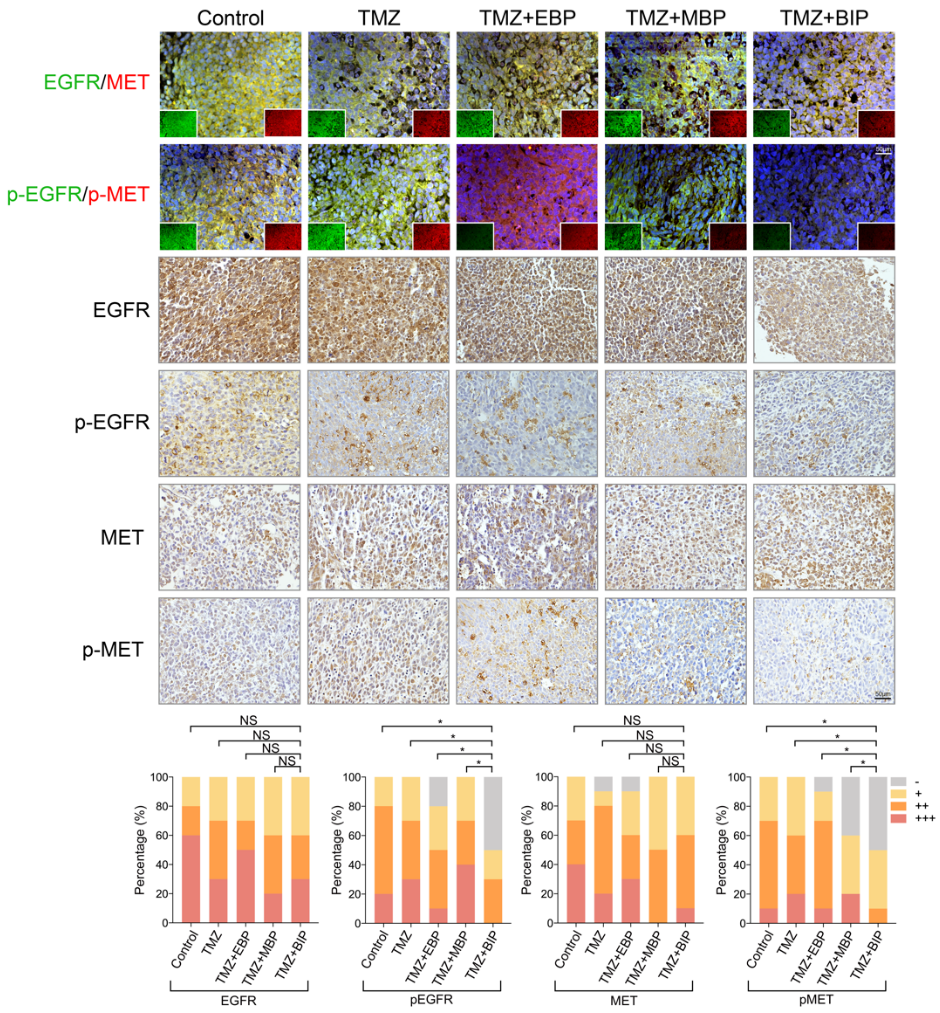

Supplementary Figure 17. The EGFR, MET, p-EGFR and p-MET expression in xenograft glioma tissues treated with TMZ and different nanoinhibitors.

The histogram represented the quantitative evaluation of the immunohistochemistry assay. Scale bar = 50 µm. P value is determined by Chi-squared test. Significant results are presented as NS non-significant, *P < 0.05.

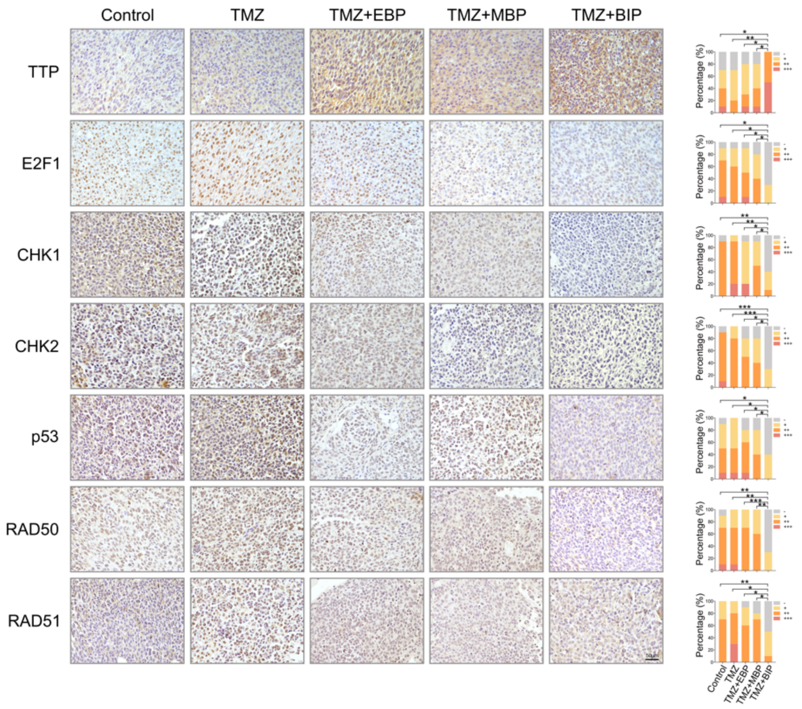

Supplementary Figure 18. The expression of TTP, E2F1 and DNA damage repair modules in xenograft glioma tissues treated with TMZ and different nanoinhibitors.

The histogram represented the quantitative evaluation of the immunohistochemistry assay. Scale bar = 50 μm. P value is determined by Chi-squared test. Significant results are presented as *P < 0.05, **P < 0.01, ***P < 0.001.

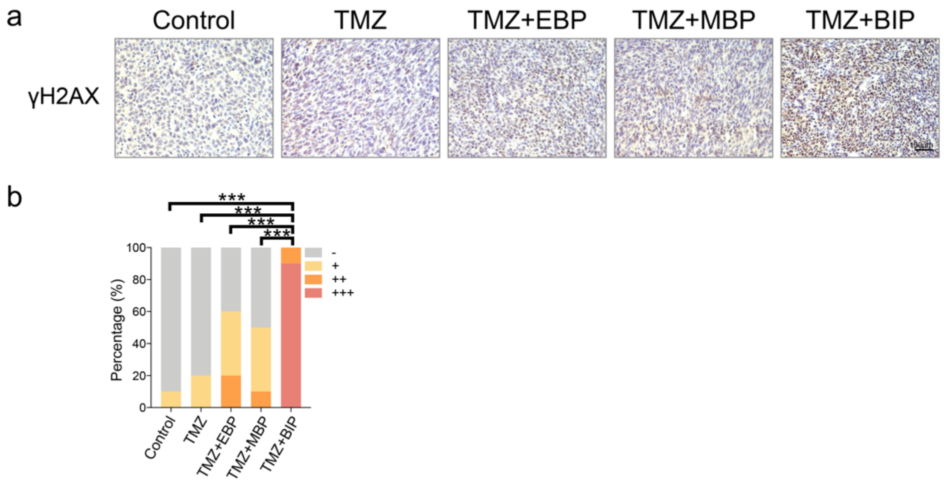

Supplementary Figure 19. The expression of γH2AX in xenograft glioma tissues treated with TMZ and different nanoinhibitors.

(a) The immunohistochemistry of γH2AX in xenograft glioma tissues treated with TMZ and different nanoinhibitors. Scale bar = 100 μm. **(b)** The histogram represents the quantitative evaluation of the immunohistochemistry assay (n = 10). P value is determined by Chi-squared test. Significant results are presented as ***$P < 0.001$

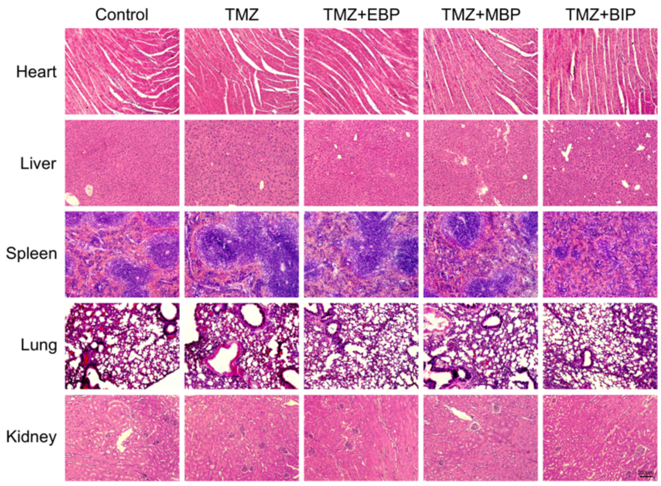

Supplementary Figure 20. The H&E staining of heart, liver, spleen, lung and kidney tissues in mice treated with TMZ and different nanoinhibitors.

The H&E staining images showed the heart, liver, spleen, lung and kidney tissues in mice treated with TMZ and different nanoinhibitors. Scale bar = 200 μm.

Figure 3a

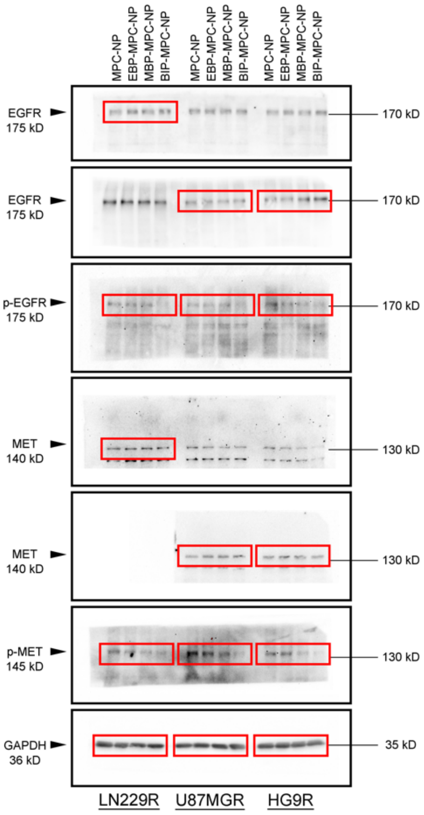

Figure 3b & Supplementary Figure 7c

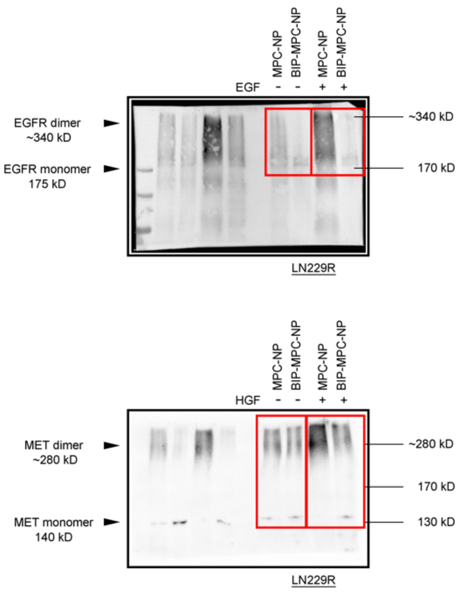

Figure 3d

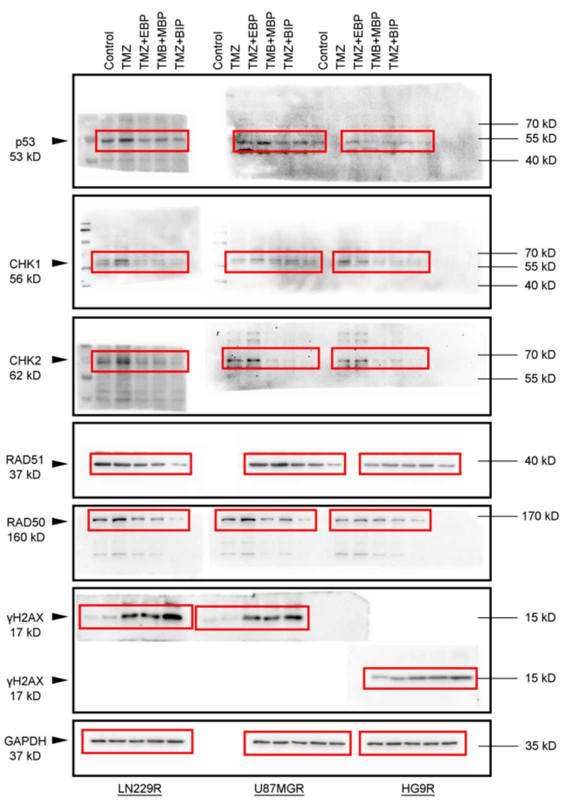

Figure 5b

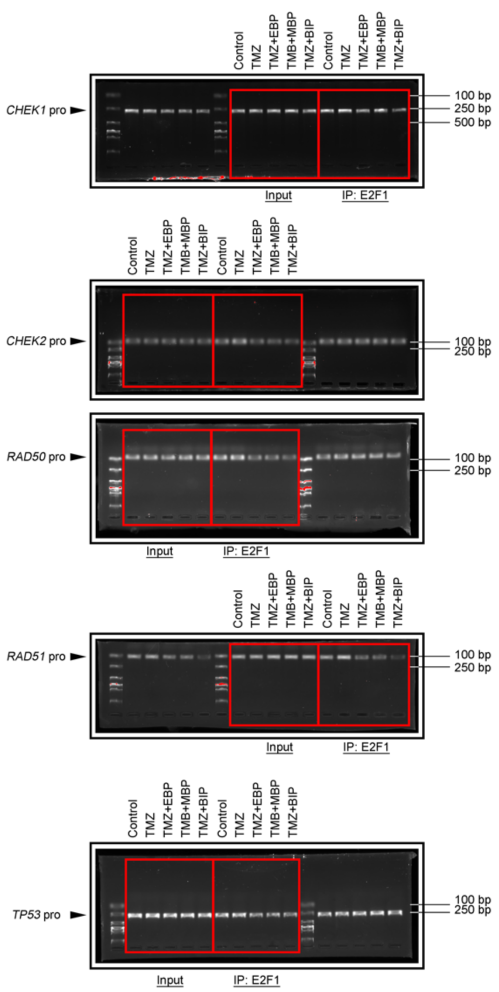

Figure 5d

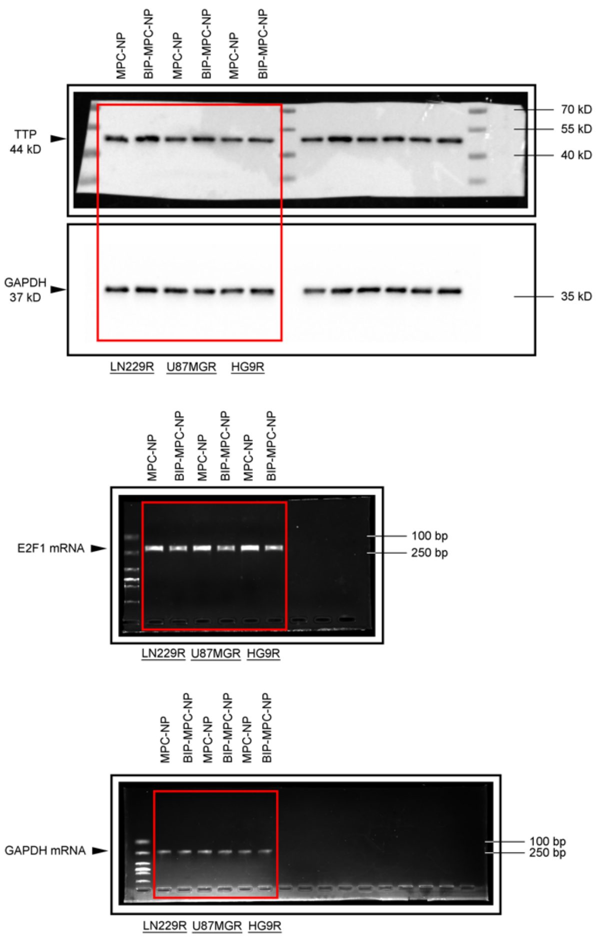

Supplementary Figure 3

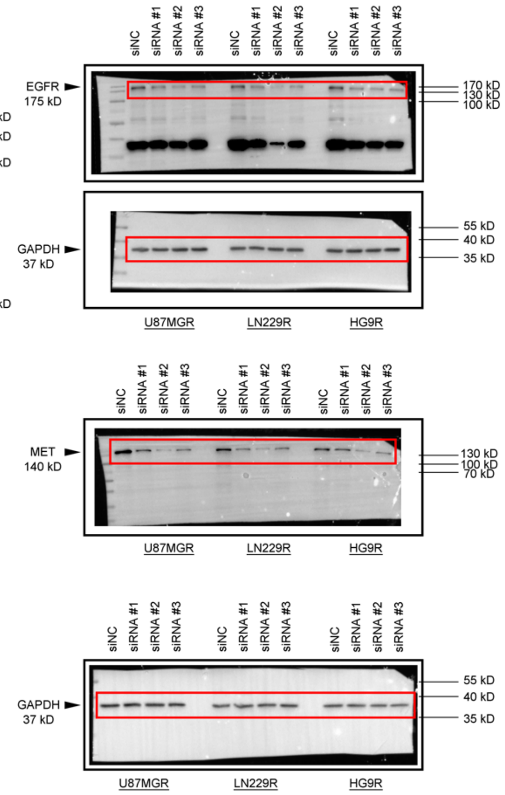

Supplementary Figure 4

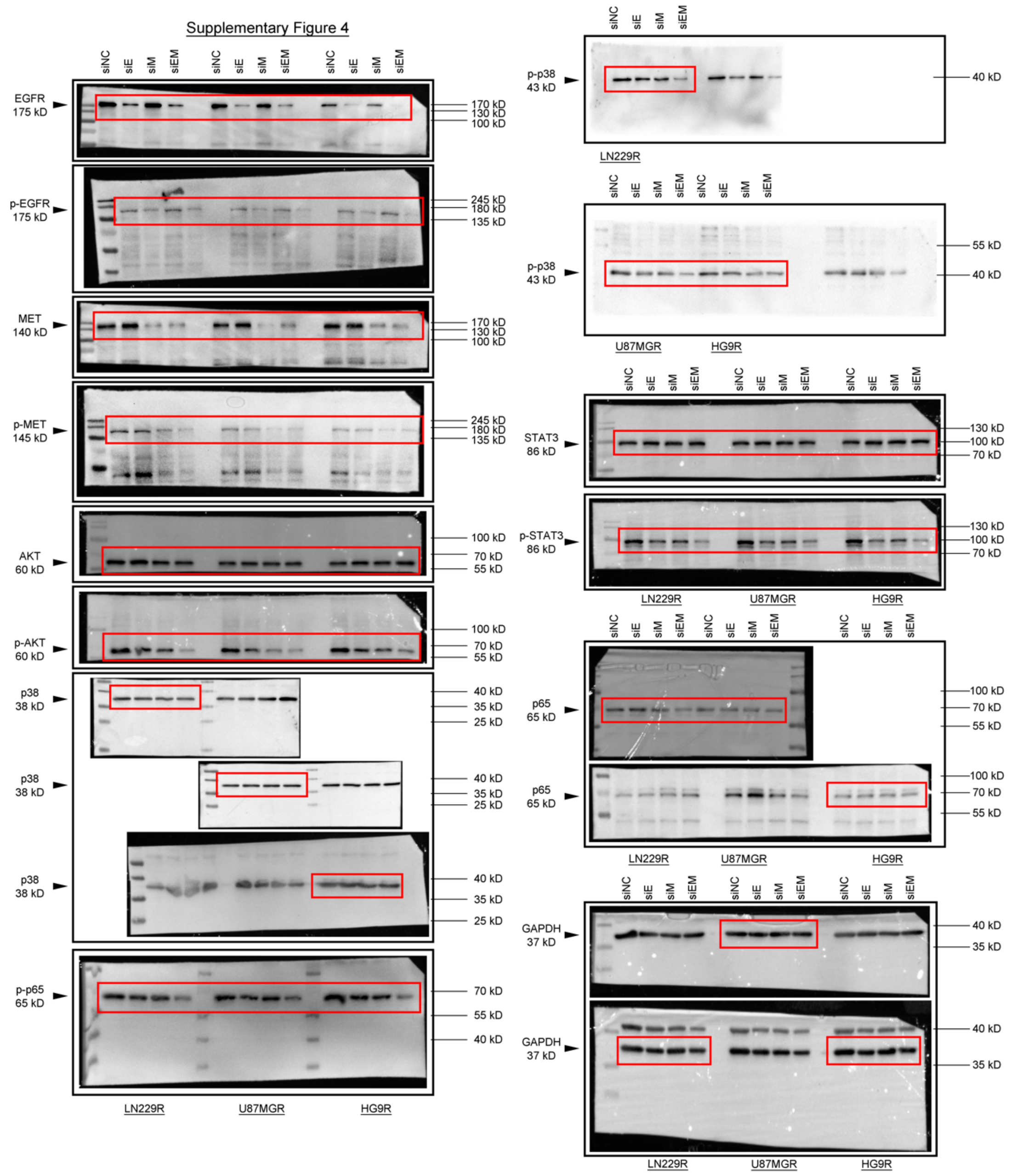

Supplementary Figure 7b & d

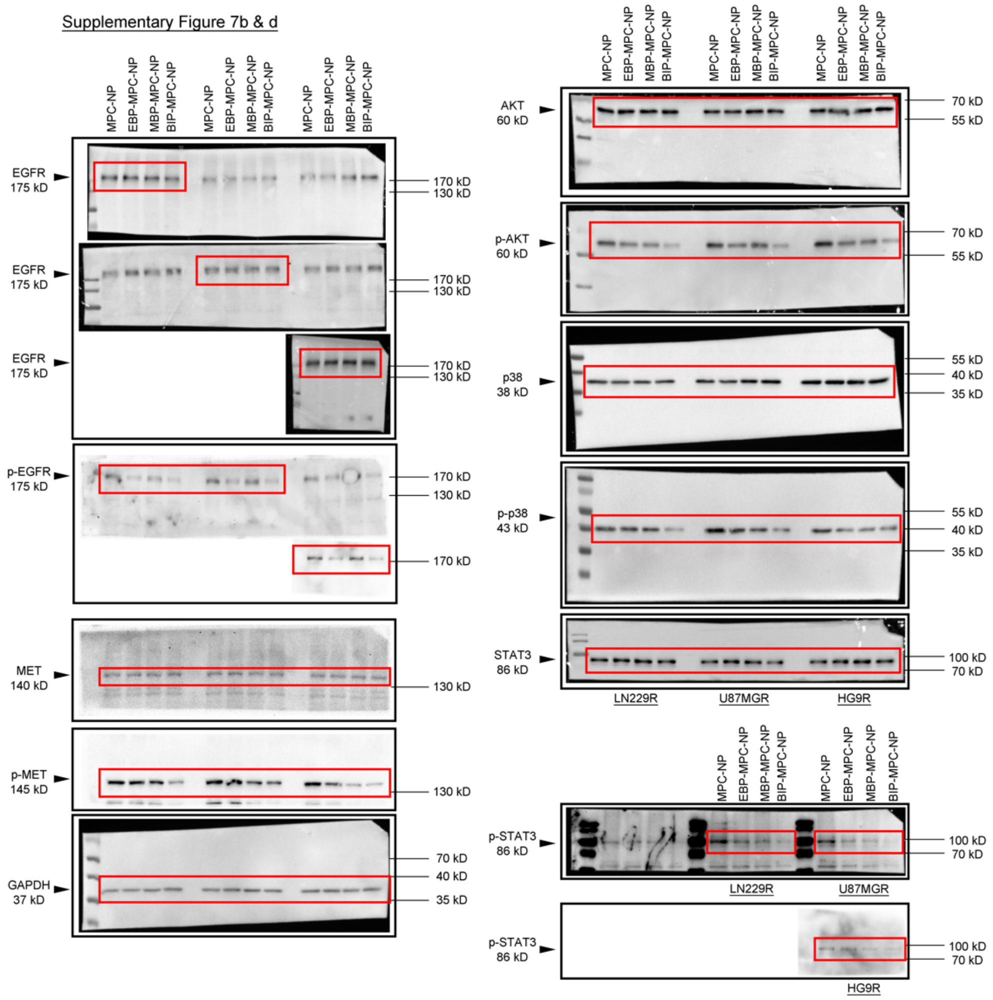

Supplementary Figure 7d

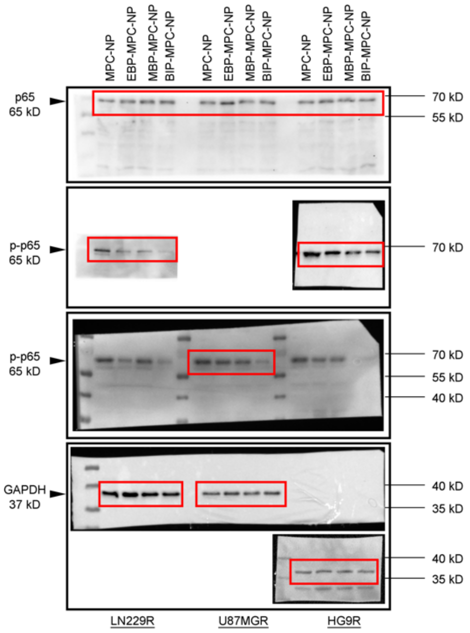

Supplementary Figure 15c

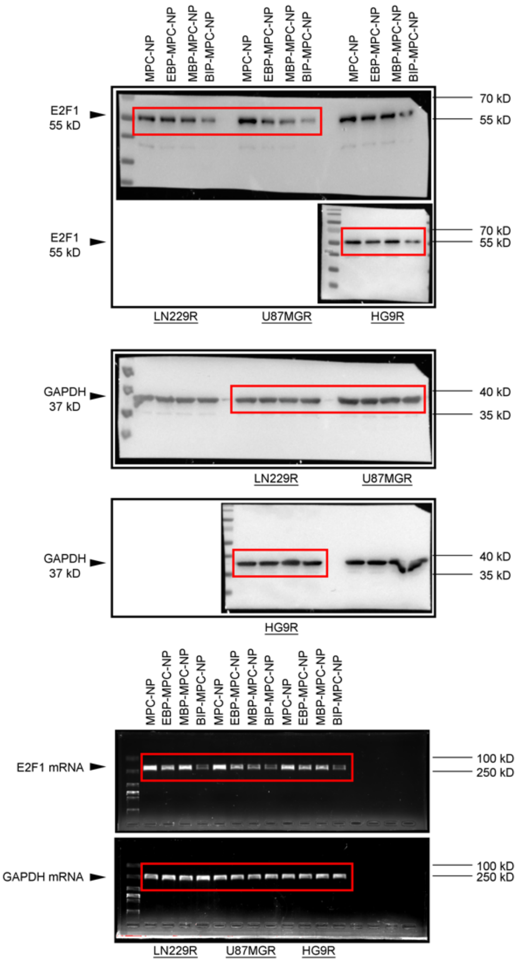

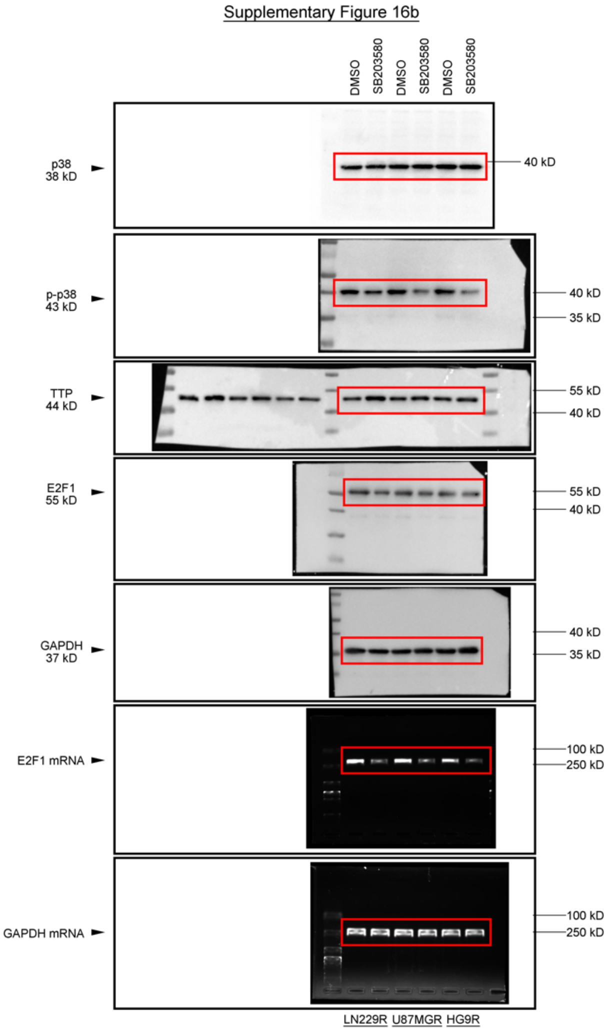

Supplementary Figure 21. Uncropped blots.

Uncropped images of all blots shown in main and supplementary figures. Molecular weight markers are indicated on the right side of each blot, whereas predicted molecular weight of the protein of interest indicated by an arrow on the left.

Supplementary Table 1. The sequences of siRNAs targeting EGFR or MET.

Target	Catalog	Sequence
EGFR	siRNA#1	5'-CACAGTGGAGCGAATTCCTTTGGAA-3'
EGFR	siRNA#2	5'-GGATCCCAGAAGGTGAGAAAGTTAA-3'
EGFR	siRNA#3	5'-CACCGTGGCTTGCATTGATAGAAAT-3'
MET	siRNA#1	5'-AGTCCGAGATGAATGTGAATATGAA-3'
MET	siRNA#2	5'-CGCTCTAATTCAGAGATAATCTGTT-3'
MET	siRNA#3	5'-CCAGTAGCCTGATTGTGCATTTCAA-3'

Supplementary Table 2. The DLS and PDI values of nanoinhibitors

	**MPC-NP**	**EBP-MPC-NP**	**MBP-MPC-NP**	**BIP-MPC-NP**
DLS (nm)	18.84 ± 1.65	24.56 ± 2.29	22.67 ± 2.20	23.65 ± 2.23
PDI	0.18± 0.02	0.26 ± 0.03	0. 20 ± 0.04	0.26 ± 0.06

Supplementary Table 3. The differential expressed proteins and the gene ontology (GO) analysis.

Protein	Gene Symbol	log_2FC	$-log_{10}P$	GO analysis
MIG6	ERRFI1	1.66	8.44	Negative_Regulation_Of_Phosphorus_Metabolic_Process//Negative_Regulation_Of_Phosphate_Metabolic_Process
CMYC	MYC	3.62	7.57	Regulation_Of_DNA_Metabolic_Process//Regulation_Of_Anatomical_Structure_Morphogenesis
CD31	PECAM1	3.89	6.95	Signal_Transduction//Cellular_Membrane_Organization//Cellular_Membrane_Organization
CYCLIND1	CCND1	1.80	6.93	Signal_Transduction//Response_To_UV//Response_To_Steroid_Hormone_Stimulus//DNA_Damage_Response__Signal_Transduction//Mammary_Gland_Development //DNA_Integrity_Checkpoint//DNA_Damage_Checkpoint
BECLIN	BECN1	0.89	6.61	Cellular_Component_Biogenesis//Response_To_Starvation
CD49B	ITGA2	1.91	6.20	Mammary_Gland_Development//Response_To_Mechanical_Stimulus
BCLXL	BCL2L1	1.97	6.15	Cellular_Membrane_Organization//Cellular_Membrane_Organization//Ovulation_Cycle//Ovulation_Cycle_Process
GATA3	GATA3	1.57	5.64	Response_To_Steroid_Hormone_Stimulus
CMETPY1235	MET	1.29	5.17	Signal_Transduction//Phosphorylation//Transmembrane_Receptor_Protein_Tyrosine_Kinase_Activity
CHK1	CHEK1	1.18	5.16	Signal_Transduction//Phosphorylation//DNA_Repair//DNA_Damage_Response__Signal_Transduction//DNA_Integrity_Checkpoint//DNA_Damage_Checkpoint
CDK1	CDK1	1.71	5.07	Signal_Transduction//Cellular_Component_Biogenesis//Phosphorylation//Regulation_Of_DNA_Metabolic_Process//DNA_Damage_Response__Signal_Transduction// DNA_Integrity_Checkpoint//DNA_Damage_Checkpoint//Positive_Regulation_Of_DNA_Metabolic_Process//Regulation_Of_DNA_Replication//Regulation_Of_Protei n_Import_Into_Nucleus
XRCC1	XRCC1	1.19	4.83	DNA_Repair
CLAUDIN7	CLDN7	1.60	4.65	Cell_Cell_Junction
SMAD4	SMAD4	1.87	4.52	Cellular_Component_Biogenesis//Signal_Transduction//Regulation_Of_Anatomical_Structure_Morphogenesis//Regulation_Of_Protein_Import_Into_Nucleus
EGFR	EGFR	3.13	4.44	Signal_Transduction//Phosphorylation//Cellular_Membrane_Organization//Cellular_Membrane_Organization//Double_Stranded_DNA_Binding//Gland_Morphogenesis //Ovulation_Cycle//Phosphatase_Binding//Regulation_Of_Protein_Import_Into_Nucleus//Transmembrane_Receptor_Protein_Tyrosine_Kinase_Activity//Response_To_ UV
MRE11	MRE11A	1.49	4.35	Regulation_Of_DNA_Metabolic_Process//DNA_Repair//Regulation_Of_DNA_Replication//Double_Stranded_DNA_Binding//Double_Strand_Break_Repair
RAD51	RAD51	3.28	4.04	Cellular_Component_Biogenesis//Regulation_Of_DNA_Metabolic_Process//DNA_Repair//Double_Stranded_DNA_Binding//Positive_Regulation_Of_DNA_Metaboli c_Process//Double_Strand_Break_Repair
FIBRONECTIN	FN1	2.02	3.87	Regulation_Of_Anatomical_Structure_Morphogenesis
P27PT157	IFI27	0.72	3.78	Activation_Of_Caspase_Activity
1433EPSILON	YWHAE	0.93	3.73	Negative_Regulation_Of_Phosphorus_Metabolic_Process//Negative_Regulation_Of_Phosphate_Metabolic_Process
BID	BID	2.71	3.72	Cellular_Membrane_Organization//Cellular_Membrane_Organization
YB1	YBX1	1.49	3.34	Double_Stranded_DNA_Binding
HER2PY1248	ERBB2	5.37	3.13	Signal_Transduction//Phosphorylation//Regulation_Of_Anatomical_Structure_Morphogenesis//Mammary_Gland_Development//Response_To_Steroid_Hormone_Stim ulus//Gland_Morphogenesis//Phosphatase_Binding//Transmembrane_Receptor_Protein_Tyrosine_Kinase_Activity//Ovulation_Cycle_Process//Ovulation_Cycle
IRS1	IRS1	1.35	3.08	Signal_Transduction//Cellular_Component_Biogenesis//Mammary_Gland_Development//Transmembrane_Receptor_Protein_Tyrosine_Kinase_Activity
PCNA	PCNA	1.02	3.06	Signal_Transduction//Regulation_Of_DNA_Metabolic_Process//DNA_Repair//Double_Stranded_DNA_Binding//Regulation_Of_DNA_Replication
INPP4B	INPP4B	1.15	3.02	Signal_Transduction
CASPASE7CLE AVEDD198	CASP7	1.85	3.01	Response_To_UV

LKB1	STK11	0.65	2.94	Phosphorylation
PR	PGR	0.27	2.90	Signal_Transduction//Mammary_Gland_Development//Gland_Morphogenesis//Ovulation_Cycle_Process//Ovulation_Cycle
P90RSKPT359S363	TFRC	1.49	2.67	Cellular_Membrane_Organization//Cellular_Membrane_Organization
P53	TP53	2.13	2.65	Signal_Transduction//Cellular_Component_Biogenesis//Regulation_Of_DNA_Metabolic_Process//Cellular_Membrane_Organization//DNA_Repair//Cellular_Membrane_Organization//DNA_Damage_Response__Signal_Transduction//Double_Stranded_DNA_Binding//Regulation_Of_DNA_Replication//DNA_Integrity_Checkpoint//DNA_Damage_Checkpoint//Double_Strand_Break_Repair//Activation_Of_Caspase_Activity//Response_To_Starvation//Phosphatase_Binding//Response_To_UV
SHCPY317	SHC1	2.99	2.61	Signal_Transduction//Regulation_Of_DNA_Metabolic_Process//Phosphorylation//Positive_Regulation_Of_DNA_Metabolic_Process//Regulation_Of_DNA_Replication
SRCPY416	SRC	4.18	2.61	Signal_Transduction//Cellular_Component_Biogenesis//Phosphorylation
HSP70	HSPA4	6.75	2.58	Cellular_Component_Biogenesis//Cellular_Membrane_Organization//Cellular_Membrane_Organization
EGFRPY1068	EGFR	10.15	2.57	Signal_Transduction//Phosphorylation//Cellular_Membrane_Organization//Cellular_Membrane_Organization//Double_Stranded_DNA_Binding//Gland_Morphogenesis//Ovulation_Cycle//Phosphatase_Binding//Regulation_Of_Protein_Import_Into_Nucleus//Transmembrane_Receptor_Protein_Tyrosine_Kinase_Activity//Response_To_UV
CHK1PS345	CHEK1	0.44	2.51	Signal_Transduction//Phosphorylation//DNA_Repair//DNA_Damage_Response__Signal_Transduction//DNA_Integrity_Checkpoint//DNA_Damage_Checkpoint
BAK	BAK1	3.33	2.47	Cellular_Membrane_Organization//Cellular_Membrane_Organization
FOXO3A	FOXO3	2.68	2.39	Ovulation_Cycle_Process//Ovulation_Cycle
NCADHERIN	CDH2	1.14	2.37	Cellular_Component_Biogenesis//Regulation_Of_Anatomical_Structure_Morphogenesis//Cell_Cell_Junction//Phosphatase_Binding
CJUNPS73	JUN	1.39	2.36	Signal_Transduction//Negative_Regulation_Of_Phosphorus_Metabolic_Process//Negative_Regulation_Of_Phosphate_Metabolic_Process//Response_To_Starvation//Positive_Regulation_Of_DNA_Metabolic_Process//Response_To_Mechanical_Stimulus//Regulation_Of_DNA_Metabolic_Process//Double_Stranded_DNA_Binding//Regulation_Of_DNA_Replication
EGFRPY1173	EGFR	11.55	2.34	Signal_Transduction//Phosphorylation//Cellular_Membrane_Organization//Cellular_Membrane_Organization//Double_Stranded_DNA_Binding//Gland_Morphogenesis//Ovulation_Cycle//Phosphatase_Binding//Regulation_Of_Protein_Import_Into_Nucleus//Transmembrane_Receptor_Protein_Tyrosine_Kinase_Activity//Response_To_UV
ECADHERIN	CDH1	5.56	2.31	Signal_Transduction//Cellular_Component_Biogenesis//DNA_Repair//Regulation_Of_Anatomical_Structure_Morphogenesis//Cell_Cell_Junction//Regulation_Of_Protein_Import_Into_Nucleus//DNA_Damage_Response__Signal_Transduction//DNA_Integrity_Checkpoint//Phosphatase_Binding//DNA_Damage_Checkpoint//Gland_Morphogenesis
RAD50	RAD50	6.15	2.22	Regulation_Of_DNA_Metabolic_Process//DNA_Repair//Double_Strand_Break_Repair
LCK	LCK	0.90	2.19	Phosphorylation//Activation_Of_Caspase_Activity
ERALPHA	ESR1	2.76	2.14	Signal_Transduction//Regulation_Of_Anatomical_Structure_Morphogenesis//Mammary_Gland_Development//Response_To_Steroid_Hormone_Stimulus//Gland_Morphogenesis//Ovulation_Cycle_Process//Ovulation_Cycle
PCADHERIN	CDH3	0.58	1.96	Cell_Cell_Junction
P27	IFI27	1.15	1.94	Activation_Of_Caspase_Activity
BAX	BAX	0.73	1.74	Cellular_Component_Biogenesis//Regulation_Of_Anatomical_Structure_Morphogenesis//Cellular_Membrane_Organization//Cellular_Membrane_Organization//Activation_Of_Caspase_Activity
CHK2PT68	CHEK2	0.96	1.72	Signal_Transduction//Phosphorylation//DNA_Damage_Response__Signal_Transduction//DNA_Integrity_Checkpoint//DNA_Damage_Checkpoint
IGFBP2	IGFBP2	1.50	1.57	Signal_Transduction//Response_To_Mechanical_Stimulus//Response_To_Steroid_Hormone_Stimulus
ACCPS79	BMS1	1.42	1.56	Cellular_Component_Biogenesis

CAVEOLIN1	CAV1	2.22	1.44	Signal_Transduction//Cellular_Component_Biogenesis//Negative_Regulation_Of_Phosphorus_Metabolic_Process//Negative_Regulation_Of_Phosphate_Metabolic_Process//Mammary_Gland_Development//Cellular_Membrane_Organization//Cellular_Membrane_Organization//Response_To_Mechanical_Stimulus//Response_To_Starvation//Response_To_Steroid_Hormone_Stimulus//Gland_Morphogenesis
SRC	SRC	0.63	1.43	Signal_Transduction//Cellular_Component_Biogenesis//Phosphorylation
YAP	YAP1	1.03	1.33	Signal_Transduction
PAXILLIN	PXN	0.84	1.32	Signal_Transduction//Cellular_Component_Biogenesis

Supplementary Table 4. Blood routine analysis in blood samples of mice bearing orthotopic glioma at the end of experiment.

Group	WBC (K/μL)	RBC (M/μL)	Hbg (g/L)	Plt ($\times 10^9$/L)
Control	8.41±0.11	10.21±0.33	146.31±2.21	1060.98±33.29
TMZ	8.87±0.29	10.90±0.21	141.01±0.30	982.52±66.61
TMZ+EBP	9.08±0.21	9.93±0.26	151.22±1.29	990.35±56.98
TMZ+MBP	7.95±0.21	9.92±0.28	140±0.91	1103.25±59.01
TMZ+BIP	8.93±0.16	10.18±0.29	143.12±1.95	977.69±51.40

TMZ+EBP: TMZ combined with EBP-MPC-NP; TMZ+MBP: TMZ combined with MBP-MPC-NP; TMZ+BIP: TMZ combined with BIP-MPC-NP. Data was shown as mean±S.D. N = 10.

Supplementary Table 5. Values of serum enzymes in blood samples of mice treated with nanoparticles.

Group	ALT (U/L)	AST (U/L)	BUN (mmol/L)	CREA (μmol/L)	TBIL (μmol/L)
Control	43.5±1.18	231.6±1.86	8.13±0.32	11.17±0.3	2.19±0.4
TMZ	45.9±2.10	232.3±3.77	8.23±0.22	11.23±0.38	2.84±0.53
TMZ+EBP	43.4±2.70	234±3.50	8.36±0.24	11.6±0.35	2.28±0.66
TMZ+MBP	46±2.02	231.8±1.62	8.55±0.33	11.99±0.41	2.48±0.33
TMZ+BIP	47.9±2.03	237.5±4.59	8.38±0.24	11.15±0.36	2.79±0.42

TMZ+EBP: TMZ combined with EBP-MPC-NP; TMZ+MBP: TMZ combined with MBP-MPC-NP; TMZ+BIP: TMZ combined with BIP-MPC-NP. Data was shown as mean±S.D. N = 10.

Supplementary Table 6. The primers used for PCR and qPCR.

Target		Sequence	Appilication
CHEK1 pro	Forward Primer	GAGTCCCAGCCCTTCCTTTC	PCR
	Reserve Primer	AAATTCTGCCCTCCTCAGGC	
CHEK2 pro	Forward Primer	GACCCAGGAGTGGTAGGTCT	PCR
	Reserve Primer	GCCAGCTTTACCTCTCCACA	
RAD50 pro	Forward Primer	CCTGAAGCAGTCTCGGAAGG	PCR
	Reserve Primer	TCCCGGTATCGTGGGATTCT	
RAD51 pro	Forward Primer	GGCGAAAACACAAGTGGACC	PCR
	Reserve Primer	CCGCTCTGATCTCGGACTTC	
TP53 pro	Forward Primer	GCGCTTCTCGCCAAGATAGA	PCR
	Reserve Primer	AGCGGACGCCAATTCTTTTG	
E2F1 mRNA	Forward Primer	CACTTTCGGCCCTTTTGCTC	PCR/qPCR
	Reserve Primer	GATTCCCCAGGCTCACCAAA	
GAPDH mRNA	Forward Primer	GAAGGTGAAGGTCGGAGTC	PCR/qPCR
	Reserve Primer	GAAGATGGTGATGGGATTTC	

Supplementary Table 7. The antibodies used for Western blot, Immunohistochemistry, Immunofluorescence and ChIP.

Primary antibodies			
**Target**	**Cat.**	**Manufactory**	**Application (Concentration)**
EGFR	ab52894	Abcam	Western blot (1:1000); Immunohistochemistry (1:100); Immunofluorescence (1:200)
p-EGFR	S.684.2	ThermoFisher	Immunofluorescence (1:200)
p-EGFR	#3777	Cell signaling technology	Western blot (1:1000); Immunohistochemistry (1:100)
MET	A10199	Abclonal	Immunohistochemistry (1:100); Immunofluorescence (1:200)
MET	#8198	Cell signaling technology	Western blot (1:1000)
p-MET	#3077	Cell signaling technology	Western blot (1:1000); Immunohistochemistry (1:100); Immunofluorescence (1:200)
AKT	#2920	Cell signaling technology	Western blot (1:1000)
p-AKT	#4060	Cell signaling technology	Western blot (1:1000)
p38	14064-1-AP	Proteintech	Western blot (1:1000)
p-p38	#4511	Cell signaling technology	Western blot (1:1000)
STAT3	#9139	Cell signaling technology	Western blot (1:1000)
p-STAT3	#9145	Cell signaling technology	Western blot (1:1000)
p65	#8242	Cell signaling technology	Western blot (1:1000)
p-p65	#3033	Cell signaling technology	Western blot (1:1000)
CHEK1	A7653	Abclonal	Western blot (1:1000); Immunohistochemistry (1:100); Immunofluorescence (1:200)
CHEK2	#3440	Cell signaling technology	Western blot (1:1000); Immunohistochemistry (1:100); Immunofluorescence (1:200)
RAD50	ab89	Abcam	Western blot (1:1000); Immunohistochemistry (1:100); Immunofluorescence (1:200)
RAD51	ab133534	Abcam	Western blot (1:1000); Immunohistochemistry (1:100); Immunofluorescence (1:200)
TP53	#2527	Cell signaling technology	Western blot (1:1000); Immunohistochemistry (1:100); Immunofluorescence (1:200)
TTP	12737-1-AP	Proteintech	Western blot (1:1000)
E2F1	12171-1-AP	Proteintech	Western blot (1:1000)
E2F1	ab4070	Abcam	ChIP-seq (5μg in total)
γH2AX	ab2893	Abcam	Western blot (1:1000); Immunohistochemistry (1:100)

GAPDH	60004-1-Ig	Proteintech	Western blot (1:1000)

Secondary antibodies

**Target**	**Cat.**	**Manufactory**	**Application (Concentration)**
HRP-conjugated Affinipure Goat Anti-Rabbit IgG(H+L)	SA00001-2	Proteintech	Western blot (1:4000)
HRP-conjugated Affinipure Goat Anti-mouse IgG(H+L)	ZDR-5307	ZSGB-BIO	Western blot (1:4000)
Multimeric anti-rabbit/mouse IgG-HRP	SV0004	BOSTER	Immunohistochemistry
Goat anti-Rabbit IgG (H+L) Highly Cross-Adsorbed Secondary Antibody, Alexa Fluor 488	A11034	ThermoFisher	Immunofluorescence (1:1000)
Goat anti-Rabbit IgG (H+L) Highly Cross-Adsorbed Secondary Antibody, Alexa Fluor 594	A11037	ThermoFisher	Immunofluorescence (1:1000)
Goat anti-Mouse IgG (H+L) Highly Cross-Adsorbed Secondary Antibody, Alexa Fluor 488	A11029	ThermoFisher	Immunofluorescence (1:1000)
Goat anti-Mouse IgG (H+L) Highly Cross-Adsorbed Secondary Antibody, Alexa Fluor 594	A11032	ThermoFisher	Immunofluorescence (1:1000)
