# Supplementary material for: Dual functionalized brain-targeting nanoinhibitors restrain temozolomide-resistant glioma via attenuating EGFR and MET signaling pathways
Source: Nat Commun. 2020 Jan 30;11:594. doi: 10.1038/s41467-019-14036-x (PMC6992617; doi:10.1038/s41467-019-14036-x)
Supplement: Supplementary file 3 — Description of Additional Supplementary Files [file 41467_2019_14036_MOESM3_ESM.pdf]

Description of Additional Supplementary Files

File Name: Supplementary Data 1
Description: The CNV data of RTK genes in LGG and GBM.

File Name: Supplementary Data 2
Description: The differential expressed RTK genes between LN229 and LN229R.
