# Supplementary material for: Dual functionalized brain-targeting nanoinhibitors restrain temozolomide-resistant glioma via attenuating EGFR and MET signaling pathways
Source: Nat Commun. 2020 Jan 30;11:594. doi: 10.1038/s41467-019-14036-x (PMC6992617; doi:10.1038/s41467-019-14036-x)
Supplement: Supplementary file 6 — Reporting Summary [file 41467_2019_14036_MOESM6_ESM.pdf]

nature research

Corresponding author(s): Chuanlu Jiang
Yang Liu
Jinquan Cai

Last updated by author(s): Nov 28, 2019

Reporting Summary

Nature Research wishes to improve the reproducibility of the work that we publish. This form provides structure for consistency and transparency in reporting. For further information on Nature Research policies, see Authors & Referees and the Editorial Policy Checklist .

Statistics

For all statistical analyses, confirm that the following items are present in the figure legend, table legend, main text, or Methods section.

n/a	Confirmed	
☐	☒	The exact sample size (*n*) for each experimental group/condition, given as a discrete number and unit of measurement
☐	☒	A statement on whether measurements were taken from distinct samples or whether the same sample was measured repeatedly
☐	☒	The statistical test(s) used AND whether they are one- or two-sided<br>*Only common tests should be described solely by name; describe more complex techniques in the Methods section.*
☐	☒	A description of all covariates tested
☐	☒	A description of any assumptions or corrections, such as tests of normality and adjustment for multiple comparisons
☐	☒	A full description of the statistical parameters including central tendency (e.g. means) or other basic estimates (e.g. regression coefficient) AND variation (e.g. standard deviation) or associated estimates of uncertainty (e.g. confidence intervals)
☐	☒	For null hypothesis testing, the test statistic (e.g. *F*, *t*, *r*) with confidence intervals, effect sizes, degrees of freedom and *P* value noted<br>*Give P values as exact values whenever suitable.*
☒	☐	For Bayesian analysis, information on the choice of priors and Markov chain Monte Carlo settings
☒	☐	For hierarchical and complex designs, identification of the appropriate level for tests and full reporting of outcomes
☐	☒	Estimates of effect sizes (e.g. Cohen's *d*, Pearson's *r*), indicating how they were calculated

Our web collection on statistics for biologists contains articles on many of the points above.

Software and code

Policy information about availability of computer code

Data collection	No software was used for data collection.
Data analysis	GraphPad software version 7.0 (GraphPad Software, CA, USA); IBM SPSS Statistics 23.0 (SPSS, Chicago, USA); Gene Cluster 3.0 and Gene Tree View; R version 3.5.1 (Feather Spray) and Flowjo 10 were used for data analysis and visuslization.

For manuscripts utilizing custom algorithms or software that are central to the research but not yet described in published literature, software must be made available to editors/reviewers. We strongly encourage code deposition in a community repository (e.g. GitHub). See the Nature Research guidelines for submitting code & software for further information.

Data

Policy information about availability of data

All manuscripts must include a data availability statement. This statement should provide the following information, where applicable:
- Accession codes, unique identifiers, or web links for publicly available datasets
- A list of figures that have associated raw data
- A description of any restrictions on data availability

Source data in this study are available on https://www.ncbi.nlm.nih.gov/geo/query/acc.cgi?acc=GSE113510, https://www.ncbi.nlm.nih.gov/geo/query/acc.cgi?acc=GSM2634759, http://cancergenome.nih.gov/. The authors declare that all data supporting the findings of this study are available within the paper and the Supplementary Material.

Field-specific reporting

Please select the one below that is the best fit for your research. If you are not sure, read the appropriate sections before making your selection.

Life sciences study design

All studies must disclose on these points even when the disclosure is negative.

Sample size	The choice of sample size has been reported in a large amount of literature.
Data exclusions	No data were excluded from the analyses.
Replication	All experiments were successfully repeated st least 3 times.
Randomization	No randomization was performed.
Blinding	No blinding was performed in this study.

Reporting for specific materials, systems and methods

We require information from authors about some types of materials, experimental systems and methods used in many studies. Here, indicate whether each material, system or method listed is relevant to your study. If you are not sure if a list item applies to your research, read the appropriate section before selecting a response.

Materials & experimental systems

n/a	Involved in the study
☐	☒ Antibodies
☐	☒ Eukaryotic cell lines
☒	☐ Palaeontology
☐	☒ Animals and other organisms
☒	☐ Human research participants
☒	☐ Clinical data

Methods

n/a	Involved in the study
☒	☐ ChIP-seq
☐	☒ Flow cytometry
☒	☐ MRI-based neuroimaging

Antibodies

Antibodies used

EGFR (ab52894); p-EGFR (S.684.2); p-EGFR (#3777); MET (A10199); MET (#8198); p-MET (#3077); AKT (#2920); p-AKT (#4060); p38 (14064-1-AP); p-p38 (#4511); STAT3 (#9139); p-STAT3 (#9145); p65 (#8242); p-p65 (#3033); CHEK1 (A7653); CHEK2 (#3440); RAD50 (ab89); RAD51 (ab133534); TP53 (#2527); TTP (12737-1-AP); E2F1 (12171-1-AP); E2F1 (ab4070); γH2AX (ab2893); GAPDH (60004-1-Ig).

Validation

Anti-EGFR antibody [EP38Y] (ab52894): Synthetic peptide within Human EGFR. The exact sequence is proprietary. Entrez Gene: 1956 Human;

Phospho-EGFR (Tyr1068) Monoclonal Antibody (S.684.2): Synthetic phosphopeptide corresponding to residues surrounding pTyr1068 of human EGF receptor. Entrez Gene: 1956 Human;

Phospho-EGF Receptor (Tyr1068) (D7A5) XP® Rabbit mAb #3777: Phospho-EGF Receptor (Tyr1068) (D7A5) XP® Rabbit mAb detects endogenous EGF receptor only when phosphorylated at Tyr1068. Xue, F., An, C., et al. (2019);

MET Monoclonal Antibody (A10199): Gene ID: 4233. This gene encodes a member of the receptor tyrosine kinase family of proteins and the product of the proto-oncogene MET.

Met (D1C2) XP® Rabbit mAb #8198: Met (D1C2) XP® Rabbit mAb recognizes endogenous levels of total Met protein. Entrez-Gene Id: 4233. Cooper, C.S. et al. (1984) Nature 311, 29-33.

Phospho-Met (Tyr1234/1235) (D26) XP Rabbit mAb #3077: Phospho-Met (Tyr1234/1235) (D26) XP Rabbit mAb detects endogenous levels of Met only when phosphorylated at Tyr1234/1235. Entrez-Gene Id: 4233

Akt (pan) (40D4) Mouse mAb #2920: Akt (pan) (40D4) Mouse mAb detects endogenous levels of total Akt protein. Entrez-Gene Id: 207 , 208 , 10000. Franke, T.F. et al. (1997) Cell 88, 435-7.

Phospho-Akt (Ser473) (D9E) XP Rabbit mAb #4060: Phospho-Akt (Ser473) (D9E) XP Rabbit mAb detects endogenous levels of Akt only when phosphorylated at Ser473. Burgering, B.M. and Coffer, P.J. (1995) Nature 376, 599-602.

P38 MAPK Antibody Rabbit Polyclonal 14064-1-AP: Immunogen: p38 MAPK fusion protein Ag5115. Source: Rabbit. MAPK14 (mitogen-activated protein kinase 14) is also named as SAPK2A, p38MAPK, CSBP1, RK, p38, EXIP, Mxi2, CSBP2, PRKM14, PRKM15, CSPB1, p38ALPHA and belongs to the MAP kinase subfamily. PMID: 27665476

Phospho-p38 MAPK (Thr180/Tyr182) (D3F9) XP Rabbit mAb #4511: Phospho-p38 MAPK (Thr180/Tyr182) (D3F9) XP® Rabbit mAb detects endogenous levels of p38 MAPK only when phosphorylated at Thr180 and Tyr182. This antibody does not cross-react with the phosphorylated forms of either p42/44 MAPK or SAPK/JNK. Entrez-Gene Id: 1432, 5600 , 5603 , 6300. Rouse, J. et al. (1994) Cell 78, 1027-1037.

Stat3 (124H6) Mouse mAb #9139: Stat3 (124H6) Mouse mAb detects endogenous levels of total Stat3 protein. Entrez-Gene Id: 6774. Heim, M.H. (1999) J Recept Signal Transduct Res 19, 75-120.

Phospho-Stat3 (Tyr705) (D3A7) Rabbit mAb #9145: Phospho-Stat3 (Tyr705) (D3A7) Rabbit mAb detects endogenous levels of Stat3 only when phosphorylated at tyrosine 705. Entrez-Gene Id: 6774. Heim, M.H. (1999) J Recept Signal Transduct Res 19, 75-120.

NF-κB p65 (D14E12) Rabbit mAb #8242: NF-κB p65 (D14E12) XP® Rabbit mAb recognizes endogenous levels of total NF-κB p65/RelA protein. Entrez-Gene Id: 5970. Baeuerle, P.A. and Henkel, T. (1994) Annu Rev Immunol 12, 141-79.
Phospho-NF-κB p65 (Ser536) (93H1) Rabbit mAb #3033: Phospho-NF-kappaB p65 (Ser536) (93H1) Rabbit mAb detects NF-κB p65 only when phosphorylated at Ser536. Entrez-Gene Id: 5970. Baeuerle, P.A. and Henkel, T. (1994) Annu Rev Immunol 12, 141-79.
CHEK1 Polyclonal Antibody A7653: The protein encoded by this gene belongs to the Ser/Thr protein kinase family. Gene ID: 1111.
Chk2 (1C12) Mouse mAb #3440: Chk2 (1C12) Mouse mAb detects endogenous levels of total Chk2 protein. Entrez-Gene Id: 11200. Allen, J.B. et al. (1994) Genes Dev. 8, 2401-2415.
Anti-Rad50 antibody [13B3/2C6] (ab89): Fusion protein containing the complete coding region (amino acids 1-425) of RAD50 expressed in E.coli. Entrez Gene: 10111 Human. Hwang SY et al. Nucleic Acids Res 47:9160-9179 (2019).
Recombinant Anti-Rad51 antibody [EPR4030(3)] (ab133534): Synthetic peptide within Human Rad51 aa 1-100. Entrez Gene: 5888 Human. Pond KW et al. PLoS Genet 15:e1007942 (2019). PubMed: 30735491
p53 (7F5) Rabbit mAb #2527: p53 (7F5) Rabbit mAb detects endogenous levels of total p53 protein. This antibody binding has been mapped to the amino terminus region of human p53 protein. Entrez-Gene Id: 7157. Levine, A.J. (1997) Cell 88, 323-31.
TTP Antibody Rabbit Polyclonal 12737-1-AP: Immunogen: ZFP36 fusion protein Ag3461. Synonyms G0S24, GOS24, NUP475, Protein TIS11A, RNF162A, TIS11, TIS11A, Tristetraproline, TTP, Zfp 36, ZFP36, Zinc finger protein 36 homolog. Gene ID (NCBI): 7538.
E2F1 Antibody Rabbit Polyclonal 12171-1-AP: Immunogen: E2F1 fusion protein Ag2813. Gene ID (NCBI): 1869. This antibody is a rabbit polyclonal antibody raised against the C-terminal 350 aa sequence of E2F1 protein, specifically recognizes the 47kd human E2F1 protein. The sumoylated E2F1 is bout 55 kDa.
Anti-E2F1 antibody [KH95] - ChIP Grade (ab4070): Transcription activator that binds DNA cooperatively with dp proteins through the E2 recognition site, 5'-TTTC[CG]CGC-3' found in the promoter region of a number of genes whose products are involved in cell cycle regulation or in DNA replication. Entrez Gene: 1869 Human. Zou Y et al. Mol Ther Nucleic Acids 14:550-561 (2019). PubMed: 30771617.
Anti-gamma H2A.X (phospho S139) antibody - ChIP Grade (ab2893): Synthetic peptide conjugated to KLH derived from within residues 100 - 200 of Human gamma H2A.X, phosphorylated at S139. Entrez Gene: 3014 Human Du M et al. Oncol Rep 41:1497-1508 (2019). PubMed: 30569179.
GAPDH Antibody Mouse Monoclonal 60004-1-Ig: It can recognize the 36 kDa GAPDH protein in most cells/tissues. Gene ID (NCBI): 2597. PMID: 23885286, 23877755, 19368702.

Eukaryotic cell lines

Policy information about cell lines

Cell line source(s)	LN229 and U87MG cells were purchased from the Chinese Academy of Sciences Cell Bank. Patient-derived GBM cells HG9 and were isolated from discarded GBM specimens.
Authentication	Each cell line used was authenticated.
Mycoplasma contamination	All cell lines were negative for mycoplasma contamination.
Commonly misidentified lines (See ICLAC register)	N/A

Animals and other organisms

Policy information about studies involving animals; ARRIVE guidelines recommended for reporting animal research

Laboratory animals	Four-week-old female athymic BALB/c nude mice were used.
Wild animals	N/A
Field-collected samples	N/A
Ethics oversight	We have complied with all relevant ethical regulations for animal testing and research. All animal experiments were performed according to Health guidelines of Harbin Medical University Institutional Animal Use and Care Committee.

Note that full information on the approval of the study protocol must also be provided in the manuscript.

Flow Cytometry

Plots

Confirm that:

- [x] The axis labels state the marker and fluorochrome used (e.g. CD4-FITC).
- [x] The axis scales are clearly visible. Include numbers along axes only for bottom left plot of group (a 'group' is an analysis of identical markers).
- [x] All plots are contour plots with outliers or pseudocolor plots.
- [x] A numerical value for number of cells or percentage (with statistics) is provided.

Methodology

Sample preparation

GBM cells were seeded into 12-well plates at a density of 2 ×105 cells per well in 1 mL fresh DMEM (supplemented with 10% FBS). After incubating for overnight, the culture medium of each well was replaced with 1 mL of fresh medium containing different nanoinhibitors. After 1-hour further incubation, the culture medium was removed and cells were washed three times with PBS buffer and detached by 0.02% (w/v) EDTA and 0.25% (w/v) trypsin solution, and then dispersed in 0.5 mL of PBS for flow cytometric measurement. Cells treated with PBS were used as control. Data were analyzed under FlowJo software.

Instrument

BD FACSCanto II

Software

FlowJo software

Cell population abundance

We didn't use the sorting function of flow cytomerter in the present study

Gating strategy

The cell debris and dead cells were excluded by establishing the rational preliminary FSC/SSC gates

☐ Tick this box to confirm that a figure exemplifying the gating strategy is provided in the Supplementary Information.
